# Prophylactic endovascular balloon occlusion of the aorta in cases of placenta accreta spectrum during caesarean section: points from the anaesthesiologist’s perspective

**DOI:** 10.1186/s12884-020-03136-y

**Published:** 2020-08-05

**Authors:** Haijuan Zhu, Shengyou Wang, Jingfa Shi, Lamei Yao, Li Wang, Hongbo Chen, Xiangdong Fang

**Affiliations:** 1 Department of Anaesthesiology, Anhui Women and Child Health Care Hospital, 230601 Hefei, China; 2 Department of Obstetrics and Gynecology, Anhui Women and Child Health Care Hospital, 230601 Hefei, China

**Keywords:** Placenta accreta spectrum, Prophylactic balloon occlusion, Perioperative anaesthesia management

## Abstract

**Background:**

The placenta accreta spectrum (PAS) is a severe complication of pregnancy and is associated with massive haemorrhage, hysterectomy, and even perinatal maternal-foetal death. Prophylactic abdominal aortic balloon occlusion (PAABO) is a novel and efficient therapy for these patients. The aim of this study was to investigate the benefits, potential risks, and characteristics of anaesthesia management.

**Methods:**

A total of 48 parturients with PAS were enrolled and divided into two groups. Group A (*n* = 25) received PAABO, and Group B (*n* = 23) underwent a normal operative procedure. The characteristics of the general parameters, anaesthesia, and operative procedure were noted. Data on vital signs including systolic blood pressure (SBP), diastolic blood pressure (DBP) and heart rate (HR) during the operation were recorded. Before and after the procedure, hepatic and renal function and lactate dehydrogenase (LDH) were also measured.

**Results:**

The characteristics of the groups were comparable. PAABO significantly reduced estimated blood loss, which was ≥ 1000 ml. Drastic fluctuations in SBP, DBP and HR were observed during inflation and deflation in Group B. After the operation, increased LDH and glutamic oxaloacetic transaminase (GOT) were observed in both groups, and increased glutamic-pyruvic transaminase (GTP) was observed in Group B.

**Conclusions:**

PAABO reduced perioperative blood loss and the risk of hysterectomy among parturients with PAS. Sophisticated anaesthetic management should be implemented to prevent or reduce perioperative complications and address internal disorders that are caused by massive blood loss.

## Background

The placenta accreta spectrum (PAS) is a severe complication of pregnancy and is associated with massive haemorrhage, hysterectomy, and even perinatal maternal-foetal death. The PAS can be divided into three types according to the depth of implantation: placenta accreta, placenta increta, and placenta percreta [1]. Parturients with suspected PAS are prenatally diagnosed by ultrasonography (US) or magnetic resonance imaging (MRI). A history of caesarean section (CS) is one of the main risk factors for the development of PAS. In recent decades, the incidence of CS for non-medical reasons has increased dramatically worldwide [2]. In China, the nationwide CS rate increased to 39% in 2008, with a higher rate in urban areas (64% in 2008) [3]. As a result, the incidence of PAS in parturients with second pregnancies and a history of CS has gradually increased in recent years [4]. CS is the optimal therapeutic approach for parturients with PAS, but with the traditional CS method, massive haemorrhaging and maternal death can occur. Additionally, hysterectomy is often inevitable due to life-threatening bleeding. Although this treatment reduces maternal mortality, it results in infertility, which is difficult for most Chinese patients and their families to accept [5]. Therefore, it is necessary to explore novel treatment options for reducing blood loss during caesarean delivery and preserving fertility in patients with PAS.

Radiation-guided endovascular interventional techniques for the treatment of obstetric haemorrhage have emerged over the past 30 ~ 40 years [6], including ligation of the internal iliac artery or uterine artery, endovascular temporary occlusion of the bilateral internal iliac, and occlusion of the uterine arteries [7]. However, the therapeutic effects of these techniques are limited due to the rapid recruitment of a rich collateral blood supply to the gravid uterus [6]. In recent years, endovascular prophylactic abdominal aortic balloon occlusion (PAABO) has been widely used in the treatment of non-compressible truncal haemorrhage, such as haemorrhage from trauma, an aneurysm or tumour of the chest or pelvic cavity [8], an abnormally adherent placenta [1]. Preoperative prophylactic placement of an occlusion balloon in the abdominal aorta and inflation after cord clamping can reduce uterine arterial pressure and blood loss in parturients with placenta accreta, thereby providing a clear surgical field, reducing bleeding and decreasing the likelihood of hysterectomy.

However, although several articles have reported the haemostatic effect and safety of PAABO, little is known about the perioperative changes and characteristics of anaesthesia management. In the present study, we retrospectively investigated the benefits, potential risks, and characteristics of anaesthesia management with PAABO.

## Methods

We performed a historical control study of patients with PAS from 1st March 2015 to 30th September 2016 in the Maternal and Child Care Hospital of Anhui Province. Written informed consent was obtained from each parturient before enrollment in the study according to the process of informed consent by the Institutional Ethics Committee of Anhui Women and Child Health Care Hospital. The study was approved by the Institutional Ethics Committee of Anhui Women and Child Health Care Hospital (2016(12)).

Forty-eight pregnant women were included in this trial, and the inclusion criteria were as follows:(1) clinical diagnosis of PAS by US or MRI; (2) gestational age > 31 weeks; and (3) elective or emergency caesarean delivery. The exclusion criteria were refusal by the parturient, antepartum bleeding, severe cardiac disease, aortic diseases and lack of complete information. The parturients were divided into two groups. Group A comprised 25 patients who underwent PAABO before CS. Group B comprised 23 patients who were not treated with PAABO: 10 did not undergo PAABO because they were hospitalized before October 2015, during which the PAABO technique was not available, and 13 declined the procedure.

In our pilot study, we found that compared with baseline values, the mean SBP level was elevated by 16.43 mmHg in Group A and 2.26 mmHg in Group B at T2 (1 min after balloon inflation). The standard deviations were 10.39 and 9.46, respectively. To achieve 90% power for the test, a minimum sample size was calculated by G*Power 3 [9] software as 10 patients in each group if *P* < 0.05 was deemed to indicate a statistically significant difference.

For Group A, the prophylactic abdominal aorta balloon was implemented by the radiologist in a DSA operating room. The right femoral artery was punctured using a modified Seldinger technique. A 12-F sheath was inserted from the femoral artery to the infrarenal abdominal aorta. The 8-F pigtail occlusion balloon catheter was embedded and located below the level of the renal artery.

After foetal delivery and umbilical cord clamping, the balloon was inflated with 6 ~ 15 ml 0.9% saline solution if the placenta could not spontaneously be expelled or severe bleeding was present. Complete occlusion was defined as absent pulsation of the dorsalis pedis artery and a foot oxygen saturation of 0 [10]. The longest single continuous balloon inflation time was 15 min, after which deflation was performed for 1 min. The duration of total occlusion was recorded for all patients. Once balloon inflation was achieved and a surgical field with less blood was obtained, the obstetricians set out to perform either conservative management or hysterectomy. When the operation was completed, the balloon was deflated for 1 min to check bleeding. If there was no recurrence of bleeding, the balloon remained deflating. If bleeding was observed, the balloon was re-inflated. Uterine artery embolization was carried out by the obstetrician. Subtotal hysterectomy was performed when necessary. If the placenta had penetrated the uterine wall and injured the bladder, the urologist might repair the bladder. Hysterectomy was carried out if the bleeding could not be controlled by the above operation. As soon as the operation was completed and there was no fresh uterine bleeding, the balloon catheter was pulled out, while the arterial sheath was removed after 6 h with compression bandaging of the femoral artery puncture sites.

In Group B, patients underwent CS and placenta evacuation without balloon occlusion of the abdominal aorta, followed by uterine artery ligation, local excision of the uterine wall, bladder repair, and even hysterectomy according to the placenta accreta location and depth. The intramuscular injection of uterotonic drugs ( 20 U of oxytocin and 100 µg of carbetocin or 250 µg of tromethamine) was given immediately after delivery of the baby and clamping of the umbilical cord.

Regarding the anaesthesia procedure, 24 parturients in Group A and 3 parturients in Group B were operated under general anaesthesia. General anaesthesia was performed with propofol (1.5 mg/kg), remifentanil (1.5 mg/kg), and succinylcholine (1.5 mg/kg) followed by tracheal intubation and mechanical ventilation just before skin incision. Midazolam (2 mg), sufentanil (0.3 µg/kg), and cisatracurium (0.15 mg/kg) were added after foetal delivery. Continuous pumping of propofol (6 mg/kg/h) and remifentanil (2 µg/kg/h) was performed to maintain the depth of anaesthesia. One parturient in Group A and 20 parturients in Group B were administered combined spinal and epidural anaesthesia. The patient’s vital signs, including blood oxygen saturation (SPO_2_), electrocardiogram (EKG), invasive arterial blood pressure (IBP), central venous pressure (CVP), and arterial blood gas analysis, were monitored in all parturients.

### Data collection

#### Primary outcomes

The primary outcomes were estimated blood loss (EBL), blood transfusion volume, incidence of hysterectomy, change in haemoglobin level after surgery, and vital parameters during surgery. EBL was calculated with the collection of blood in the suction bottle and the weight of the surgical swabs, subtracting the volume of amniotic fluid. Data on vital signs including systolic blood pressure (SBP), diastolic blood pressure (DBP) and heart rate (HR) were recorded at baseline (T0), 1 min before balloon inflation (T1), 1 min after balloon inflation (T2), 5 min after inflation (T3), 1 min after deflation (T4), and 5 min after deflation (T5).

#### Secondary outcomes

The secondary outcomes included the operating time from the time of skin incision to skin closure, the balloon occlusion time, maternal balloon occlusion-related complications, neonatal outcomes including Apgar scores at 1 and 5 min, and a comparison of the outcomes in general anaesthesia versus regional anaesthesia.

### Statistical analysis

Continuous variables were expressed as the mean ± standard deviation. Categorical variables are described as numbers and percentages. Data were analysed by using SPSS version 16.0. Normally distributed data, such as demographic data, EBL, change in haemoglobin levels and operating time, were analysed using independent samples *t*-tests. Abnormally distributed data, such as the volume of red blood cells transfused, were analysed using the Mann-Whitney *U* test. Categorical variables, such as hysterectomy, neurologic complications of the lower limbs, and gastrointestinal complications, were assessed using *Fisher’s* exact test. For laboratory data, paired *t*-tests were used to compare the preoperative and postoperative values, and two-sample *t*-tests were used to compare data from the two groups. Vital parameters including SBP were analysed by repeated measures ANOVA. *P* < 0.05 was considered statistically significant.

## Results

According to the guidelines for treatments of PAS, a total of 40 parturients were implanted abdominal aorta balloon prophylactically but only 25 balloons ultimately needed to be inflated. Data in this study were recorded for all 48 parturients with 25 cases of PAABO and 23 cases of non-treatment with PAABO. The demographic and obstetric characteristics of the parturients were comparable, and are shown in Table 1. The mean time from balloon inflation to deflation was 16.1 ± 6.4 min (Table 2), and the mean volume of 0.9% saline was 9.9 ± 3.4 ml (Table 2). The operations of 24 cases in Group A and 3 cases in Group B were completed under general anaesthesia.

**Table 1:** Demographic and obstetric characteristics

Characteristics	Group A (*n* = 25)	Group B (*n* = 23)	*P* value
Maternal age, y	32.7 ± 4.9	31.3 ± 6.4	0.404
Weight, kg	69.2 ± 8.5	70.9 ± 6.5	0.508
Gestational weeks, w	36.4 ± 2.3	36.4 ± 1.4	0.983
Gravidity	3.7 ± 1.4	3.9 ± 1.2	0.543
Prior cesarean deliveries	1.2 ± 0.5	1.2 ± 0.7	0.919
Type of cesarean delivery			0.091
Elective cesarean delivery	24 (96)	18 (78)	
Emergencycesarean delivery	1 (4)	5 (22)	
Type of placenta previa			0.424
Placenta previa alone	7 (28)	11 (48)	
Placenta previa with accreta	12 (48)	8 (35)	
Placenta previa with percreta	6 (24)	4 (17)	

Data expressed as mean ± SD or n (%). *SD* standard deviation

**Table 2:** Operative characteristics and outcomes of parturients

Outcomes	Group A (*n* = 25)	Group B (*n* = 23)	*P* value
Type of anesthesia			
General anesthesia	24 (96)	3 (13)	0.000
Regional anaesthesia	1 (4)	20 (87)	
Balloon occlusion time, min	16.1 ± 6.4		
Volume of saline in the balloon, ml	9.9 ± 3.4		
Operative time, min	79.2 ± 31.5	81.2 ± 37.9	0.352
Estimated blood loss, ml	1276.0 ± 945.7	1900.0 ± 1157.2	0.046
Estimated blood loss ≥ 1000 ml	12(48)	20(87)	0.006
Volume of red blood cells transfused	676.0 ± 931.5	713.0 ± 1008	0.485
Change in hemoglobin, g/L	14.9 ± 8.3	16.5 ± 8.6	0.526
Hysterectomy	2 (8)	4 (17)	0.407
Neurologic complications of lower limbs	2 (8)	0	0.490
Gastrointestinal complication	10 (40)	4 (17)	0.117
Thrombogenesis in lower limbs	0 (0)	0 (0)	
Apgar score < 7			0.941
1 min	1 (4)	2 (9)	
5 min	0	0	

Data expressed as mean ± SD or n (%). SD: standard deviation

The amount of EBL was significantly lower in Group A than in Group B (*P* = 0.046, Table 2). The number of patients with EBL ≥ 1000 ml was lower in Group A than in Group B, and this difference was statistically significant (*P* = 0.006, Table 2). No statistically significant differences in the number of units of red blood cells transfused, operative times, or changes in haemoglobin before and after surgery were observed (Table 2). Hysterectom**y** was performed in 2 cases in Group A and 4 cases in Group B (*P* = 0.407, Table 2). One neonate in Group A and 2 ones in Group B had Apgar scores < 7 at 1 min (*P* = 0.941, Table 2). All delivered foetuses had Apgar scores > 7 at 5 min.

**Fig. 1:**
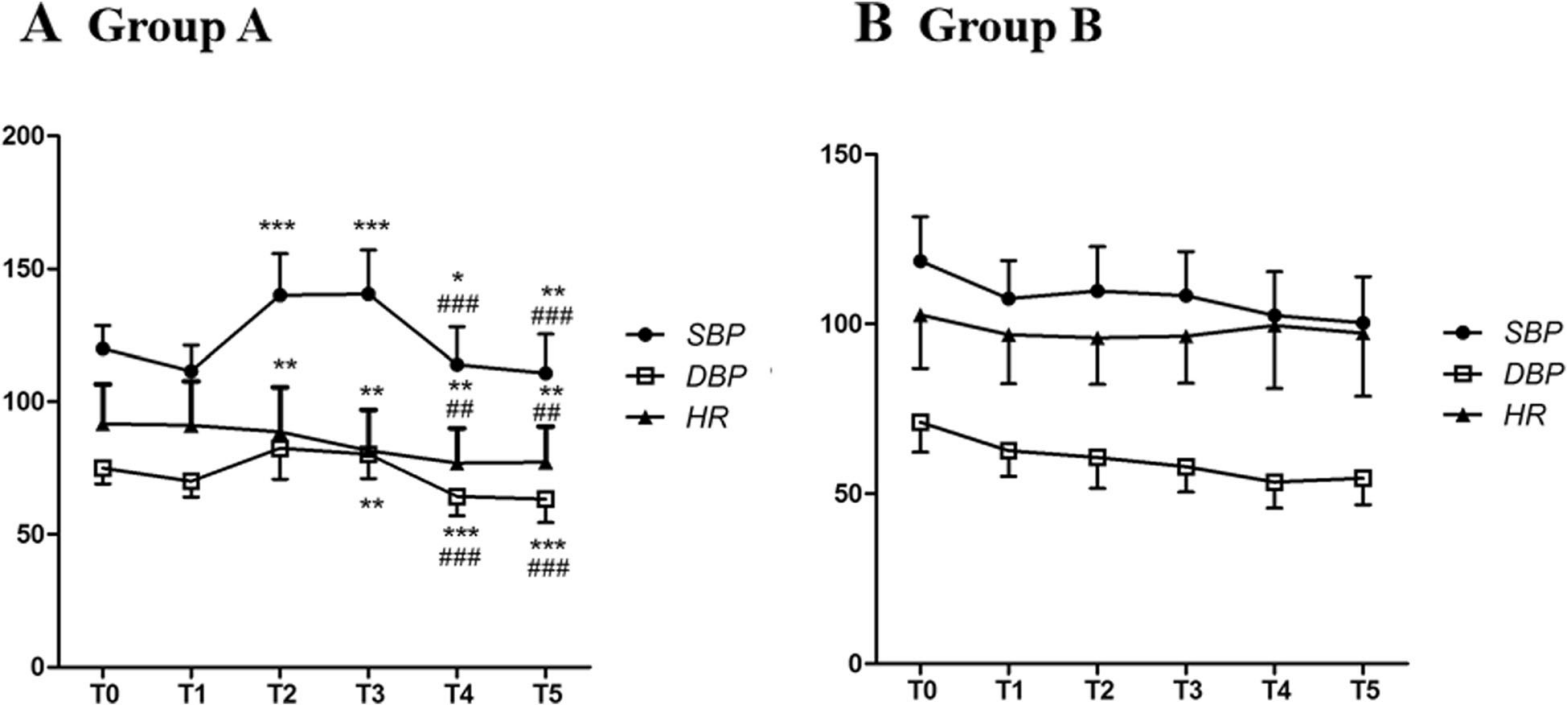
Vital signs of Group A and Group B during operation. **a **The vital sign data of the patients in Group A. **b** The vital sign data of the patients in Group (B) The data are shown as indexes of SBP, DBP and HR at different time points. T0: Baseline value, T1: 1 min before balloon inflation, T2: 1 min after balloon inflation, T3: 5 min after inflation, T4: 1 min after deflation, T5: 5 min after deflation. *P < 0.05 vs.T1, **P < 0.01 vs.T1, ***P < 0.001 vs.T1, ###*P* < 0.001 vs.T2

Data on the vital signs of the two groups were shown in Fig. 1. In Group A, there were no significant differences in SBP, DBP or HR from T0 to T1. Increased SBP, DBP and HR indexes were observed at T2 and T3 compared with T0 and T1 (*P <* 0.01, Fig. 1). Decreases in these parameters were observed at T4 and T5 compared with T0 (*P* < 0.05, Fig. 1). In Group B, there were no significant differences in SBP, DBP or HR from T1 to T5. The differences between both groups were statistically significant at T2, T3 and T4 (*P* < 0.01, Fig. 1).

Next, perioperative complications including peripheral organ dysfunction, arterial thrombosis and nerve injury of the lower limb were assessed. When the postoperative and preoperative data were compared, the index values of LDH, GPT and GOT increased significantly in both groups (*P* < 0.01, Table 3). However, no statistically significant differences were detected between the two groups (Table 3).

**Table 3:** Comparation of lab data at preoperation and postoperation in both groups

		n	Preoperation	Postoperartion	*P* Value (posto vs. preo)
LDH (IU/L)	Group A	25	174.5 ± 25.1	307.6 ± 62.9	0.000
	Group B	23	167.8 ± 36.1	322.2 ± 87.4	0.000
	*P*-value (between groups)		0.452	0.508	
GPT (IU/L)	Group A	25	14.4 ± 10.7	20.5 ± 16.0	0.005
	Group B	23	16.6 ± 14.2	20.4 ± 15.7	0.001
	*P*-value (between groups)		0.538	0.996	
GOT (IU/L)	Group A	25	18.4 ± 7.0	29.0 ± 9.8	0.000
	Group B	23	19.6 ± 9.1	30.7 ± 15.4	0.000
	*P*-value (between groups)		0.620	0.643	
Cr (µmol/L)	Group A	25	43.8 ± 9.3	44.3 ± 9.0	0.818
	Group B	23	42.8 ± 9.1	47.2 ± 9.1	0.041
	*P*-value (between groups)		0.710	0.278	
BUN (mmol/L)	Group A	25	2.9 ± 0.7	3.0 ± 1.0	0.582
	Group B	23	3.0 ± 0.8	3.1 ± 0.9	0.143
	*P*-value (between groups)		0.643	0.490	

Data expressed as mean ± SD. *SD* standard deviation; *LDH* lactate dehydrogenase; *GPT* glutamic pyruvic transaminase; *GOT* glutamic-oxaloacetic transaminase; *Cr* creatinine; *BUN* blood urea nitrogen

Gastrointestinal complications included abdominal distension, abdominal pain, diarrhoea, vomiting, paralytic intestinal obstruction (1 in Group B with bleeding volume of 1500 ml) and upper gastrointestinal stress ulcer (1 in Group A with a bleeding volume of 800 ml), which were totally found in 14 cases (10 in Group A and 4 in Group B, Table 2). 2 cases of right lower limb paraesthesia were found in Group A (Table 2), and none were recorded in Group B (Table 2). There were no cases of arterial thrombosis or maternal death (Table 2).

To compare the outcomes of mothers and babies under general anaesthesia (GA) with regional anaesthesia (RA), the time from skin incision to delivery, operative duration, EBL, and Apgar scores were collected (Table 4). The results found no significant differences between both groups in operative duration, EBL, and Apgar scores (*P* > 0.05, Table 4). However, the average duration of induction-to-delivery was 6.6 ± 2.1 min, and the average duration of skin incision to delivery was 5.4 ± 1.8 min in the GA group. The average duration of skin incision to delivery was 8.0 ± 2.7 min in the RA group, which was significantly longer than that in the GA group (*P* < 0.001, Table 4).

**Table 4:** Operative characteristics of parturients in general anesthesia and regional anesthesia

Outcomes	GA (*n* = 27)	RA (*n* = 21)	*P* value
Duration of induction-to-delivery, min	6.6 ± 2.1	/	
Duration of skin incision-to-delivery, min	5.4 ± 1.8	8.0 ± 2.7	0.000
Operative duration, min	82.6 ± 42.6	73.3 ± 18.8	0.224
Estimated blood loss, ml	1589 ± 1373	1557 ± 573.2	0.921
Apgar score at 1 min	9.0 ± 0.2	8.5 ± 1.2	0.069
Apgar score at 5 min	10.0 ± 0.2	9.9 ± 0.3	0.419
Apgar score < 7			
1 min	1(3.7)	2(9.5)	0.822
5 min	0	0	

Data expressed as mean ± SD or n (%). *SD* standard deviation; *GA* general anesthesia; *RA* regional anesthesia

## Discussion

In this study, we found that balloon occlusion could reduce EBL and the ratio of EBL ≥ 1000 ml. Here, in patients with PAS, PAABO was confirmed to have more advantages in reducing blood loss during caesarean delivery than traditional interventional therapies. These results were consistent with previous research [11].

In response to this novel technology, it is imperative for anaesthesiologists to be familiar with the physiologic changes induced by balloon inflation and deflation to conduct appropriate anaesthetic methods and to address massive haemorrhage. Until now, few reports have addressed the points about anaesthetic management. Anaesthesiologists’ goal is to develop an appropriate anaesthesia regimen with no adverse effects on women and babies, to minimize fluctuations during operative haemodynamics, to address haemorrhagic events, and to prevent perioperative complications [11].

Anaesthesia for CS with PAS remains controversial. Regarding the potential impact of anaesthesia on babies, the preferred anaesthetic method for CS is regional anaesthesia [12]. However, GA is superior to RA in patients with a risk of severe haemorrhage or prolonged surgical time. According to the previous literature, there is no significant difference in neonatal outcomes between regional and general anaesthesia [12]. In the early cases, we attempted to operate under RA but failed due to the severe discomfort of the parturients with abdominal distension at balloon inflation. Therefore, we selected GA for the remaining 24 parturients in Group A. For the parturients in Group B, we preferred RA, with 20 parturients in Group B receiving RA and 3 parturients receiving GA due to an emergency or haemorrhagic shock. In GA, the amount of anaesthetic crossing the placenta into the foetal circulation is determined by the physicochemical and structural properties of the drug as well as the dose and duration of transplacental transfer. Therefore, the duration from infusion of propofol and remifentanil to delivery of babies should be as short as possible. However, abdominal adhesion and prolonged induction-to-delivery interval might commonly occur in parturients with PAS due to a history of CS. Thus, to minimize adverse effects on babies, experienced anaesthesiologists and obstetricians as well as intimate teamwork are needed. In this study, the average duration of induction-to-delivery was 6.6 ± 2.1 min, which was longer than common CS. However, the skin incision-to-delivery interval was 5.4 ± 1.8 min in the GA group, which was significantly shorter than that in the RA group (8.0 ± 2.7 min). The difference might be attributed to the more experienced anaesthesiologists and obstetricians with intimate cooperation in the GA group. In this study, there were no differences in the Apgar scores when comparing Group A with Group B or GA with RA.

The results showed that arterial blood pressure was significantly elevated after balloon inflation, which was consistent with the report by Xin Wei [13]. A modest increase in arterial blood pressure was helpful for maintaining perfusion of the heart and brain, but excessively high pressure might be hazardous to patients with potential cardiac, pulmonary, or cerebral disease. A sudden elevation in arterial pressure would increase afterload and left ventricular work, which could lead to left ventricular dilation, myocardial ischaemia, and cerebral hyperperfusion. This might induce intracranial trauma or bleeding [14]. A recent report showed that increased blood pressure induced by balloon inflation can increase cerebral blood flow risk and exacerbate a pre-existing cerebral injury such as a contusion or intracranial haemorrhage [15]. Current guidelines recommend an optimal cerebral perfusion pressure (CPP) of 60 mmHg for patients during balloon inflation [14]. In addition, there would be a drastic reduction in blood pressure when the balloon was deflated, which might lead to ischaemia of the myocardium, brain, or intestine. Therefore, it is important for anaesthesiologists to take measures to reduce fluctuations in haemodynamics during balloon inflation and deflation. Relevant measures include increasing the depth of anaesthesia and the use of opioid analgesics and vasoactive drugs. IBP monitoring is imperative for real-time observation of vital signs and monitoring of blood gas indicators.

Complications are the most important features that anaesthesiologists need to focus on with regard to PAABO. The relevant complications include embolization of the lower extremities [7], ischaemic injury of the myocardium, kidneys, lungs, intestines and spinal cord [16] and aorta rupture or dissection, which can lead to maternal death [17]. To prevent or reduce the potential risks of PAABO, the following should be performed: (1) minimization of the occlusion time,(2) maintenance of the balloon diameter at the smallest size that blocks blood flow and (3) localization of the balloon below the renal artery origin to reduce the risk of renal injury. As currently there is no uniform standard for the duration of balloon occlusion [11]. Yeung and Wahlberg [16, 18] showed that the morbidity of postoperative renal failure decreased at < 20 min of ischaemia and increased 10-fold at > 50 min of the ischaemia. However, some studies have shown that the spinal cord might tolerate the occlusion up to 30 min [19, 20]. Andoh et al. [21] suggested a 25-min occlusion time in caesarean hysterectomy for placenta accreta. Masamoto et al. [22] suggested that the longest occlusion time be 80 min without intra- or postoperative complications. The maximum occlusion time for parturients needs to be explored. To prevent or reduce the risk of aortic rupture, the occlusion time and diameter of the balloon should both be restricted [23]. Regarding the diameter of the balloon, Liu et al.. confirmed that the diameter of the aorta could be estimated by pelvic MRI prior to surgery and that the commonly used size was 16 ~ 20 mm [1]. In this study, the longest continuous duration of balloon inflation was restricted to less than 15 min. If a continuous long-term block is needed for surgery, the balloon inflation should be alternated with 1-min deflations to restore the blood supply [17].The principle of balloon expansion in our operation was to use the minimum volume to block blood flow and limit the maximum volume to 15 ml. The mean volume for inflation of the balloon was 9.9 ± 3.4 ml. The results showed that the rate of neurologic complications of the lower limbs in Group A was 8%. Gastrointestinal complications were found in 14 cases (10 in Group A and 4 in Group B). The most common gastrointestinal dysfunction in Group B was abdominal distension, which might be a result of low intestinal movement after GA. Meanwhile, two more severe cases with paralytic intestinal obstruction (1 case in Group B) as well as upper gastrointestinal stress ulcer (1 case in Group A) might due to gastrointestinal stress response after severe bleeding. Hence, for the patients with massive bleeding, the proton pump inhibitors should be administered to reduce the stress injury to the gastrointestinal mucosa. The index values of GPT and GOT significantly increased in both groups when comparing the preoperative and postoperative indicators. The indexes were still in the normal range, suggesting that the PAABO operation did not lead to severe gastrointestinal complications. The index of postoperative LDH activity was significantly elevated in both groups, and no significant differences were found between the groups. LDH is one of most important glycolytic enzymes in cells, and it functions as a marker of ischaemia/reperfusion injury. LDH is released into the blood when cells are damaged. Haemorrhage and ischaemia/reperfusion injury can elevate LDH activity and lead to gastrointestinal/intestinal complications [24]. All of the complications and elevated biochemical indexes were resolved and returned to normal, respectively, and there was no permanent harm.

The present study had several limitations. First, it was a retrospective and single-centre study, in which there was a lack of randomness in grouping, leading to selection bias. Second, the sample size was relatively small, which might lead to a less statistically powerful sample and unstable and inaccurate results. Third, we compared the effect of GA with RA in all patients with PAS rather than those with PAABO, resulting in some defects of imbalance and incompletion. Finally, the depth of the study is insufficient, and some indexes such as lactic acid could have been affected by inflation balloon. Therefore, a prospective and multicentre study with more patients is needed.

## Conclusions

PAABO reduced perioperative blood loss and the risk of hysterectomy in parturients with PAS. Sophisticated anaesthetic management should be implemented to minimize adverse effects on mothers and babies, to prevent or treat perioperative complications, and to address gastrointestinal disorders caused by massive blood loss. The management mode of multi-disciplinary team cooperation was very necessary for successful treatment with PAS and PAABO [25].

## Data Availability

Reasonable request for anonymous data supporting the conclusions of this article should be addressed to the corresponding author.

## Abbreviations

PAS: Placenta accreta spectrum
PAABO: Prophylactic abdominal aortic balloon occlusion
CS: Caesarean section
MRI: Magnetic resonance imaging
DSA: Digital subtraction angiography
SPO_2_: Pulse oximetry
EKG: Electrocardiogram
IBP: Invasive arterial blood pressure
CVP: Venous pressure
SBP: Systolic blood pressure
DBP: Diastolic blood pressure
HR: Heart rate
SD: Standard deviation
LDH: Lactic dehydrogenase
GOT: Glutamic oxaloacetic transaminase
Cr: Creatinine
BUN: Blood urea nitrogen
GPT: Glutamic-pyruvic transaminase
CPP: Cerebral perfusion pressure

## References

[1] Liu J, Wang Y, Jiao D (2019). Prophylactic Occlusion Balloon Placement in the Abdominal Aorta Combined with Uterine or Ovarian Artery Embolization for the Prevention of Cesarean Hysterectomy Due to Placenta Accreta: A Retrospective Study. Cardiovasc Intervent Radiol.

[2] Liang H, Fan Y, Zhang N (2018). Women’s cesarean section preferences and influencing factors in relation to China’s two-child policy: a cross-sectional study. Patient Prefer Adherence.

[3] Deng W, Klemetti R, Long Q (2014). Cesarean section in Shanghai: women’s or healthcare provider’s preferences?. BMC Pregnancy Childbirth.

[4] Ying P, Lai J, Cheng P (2020). The application of prophylactic balloon occlusion of the internal iliac artery for the treatment of placenta accreta spectrum with placenta previa: a retrospective case control study. BMC Pregnancy Childbirth.

[5] Chu Q, Shen D, He L (2017). Anesthetic management of cesarean section in cases of placenta accreta, with versus without abdominal aortic balloon occlusion: study protocol for a randomized controlled trial. Trials.

[6] Wu Q, Liu Z, Zhao X (2016). Outcome of Pregnancies After Balloon Occlusion of the Infrarenal Abdominal Aorta During Caesarean in 230 Patients With Placenta Praevia Accreta. Cardiovasc Intervent Radiol.

[7] Li N, Yang T, Liu C (2018). Feasibility of Infrarenal Abdominal Aorta Balloon Occlusion in Pernicious Placenta Previa Coexisting with Placenta Accrete. Biomed Res Int..

[8] Sridhar S, Gumbert SD, Stephens C (2017). Resuscitative Endovascular Balloon Occlusion of the Aorta: Principles, Initial Clinical Experience, and Considerations for the Anesthesiologist. Anesth Analg.

[9] Faul F, Erdfelder E, Lang AG (2007). G*Power 3: a flexible statistical power analysis program for the social, behavioral, and biomedical sciences. Behav Res Methods.

[10] Duan XH, Wang YL, Han XW (2015). Caesarean section combined with temporary aortic balloon occlusion followed by uterine artery embolisation for the management of placenta accreta. Clin Radiol.

[11] Chen L, Wang X, Wang H (2019). Clinical evaluation of prophylactic abdominal aortic balloon occlusion in patients with placenta accreta: a systematic review and meta-analysis. BMC Pregnancy Childbirth.

[12] Kocaoglu N, Gunusen I, Karaman S (2012). Management of anesthesia for cesarean section in parturients with placenta previa with/without placenta accreta: a retrospective study. Ginekologia polska.

[13] Wei X, Zhang J, Chu Q (2016). Prophylactic abdominal aorta balloon occlusion during caesarean section: a retrospective case series. Int J Obstet Anesth.

[14] Carney N, Totten AM, O’Reilly C (2017). Guidelines for the Management of Severe Traumatic Brain Injury, Fourth Edition. Neurosurgery.

[15] Uchino H, Tamura N, Echigoya R, et al. “REBOA” - Is it Really Safe? A Case with Massive Intracranial Hemorrhage Possibly due to Endovascular Balloon Occlusion of the Aorta (REBOA). Am J Case Rep. 2016;17:810–3. 10.12659/AJCR.900267 PMC5091201 27799653

[16] Yeung KK, Groeneveld M, Lu JJ (2016). Organ protection during aortic cross-clamping. Best Pract Res Clin Anaesthesiol.

[17] Liu J, Li Y, Han X (2019). Comment on Y Wei etal: Comparison of Efficacy between Internal Iliac Artery and Abdominal Aorta Balloon Occlusions in Pernicious Placenta Previa Patients with Placenta Accrete. Gynecol Obstet Invest.

[18] Wahlberg E, Dimuzio PJ, Stoney RJ (2002). Aortic clamping during elective operations for infrarenal disease: The influence of clamping time on renal function. J Vasc Surg.

[19] Katz NM, Blackstone EH, Kirklin JW (1981). Incremental risk factors for spinal cord injury following operation for acute traumatic aortic transection. J Thorac Cardiovasc Surg.

[20] Papakostas JC, Matsagas MI, Toumpoulis IK (2006). Evolution of spinal cord injury in a porcine model of prolonged aortic occlusion. J Surg Res.

[21] Andoh S, Mitani S, Nonaka A (2011). Use of temporary aortic balloon occlusion of the abdominal aorta was useful during cesarean hysterectomy for placenta accreta. Masui.

[22] Masamoto H, Uehara H, Gibo M (2009). Elective use of aortic balloon occlusion in cesarean hysterectomy for placenta previa percreta. Gynecol Obstet Invest.

[23] Matsubara S. Comment on “Discussion on the Timing of Balloon Occlusion of the Abdominal Aorta during a Caesarean Section in Patients with Pernicious Placenta Previa Complicated with Placenta Accreta”. Biomed Res Int. 2018;9493878. 10.1155/2018/9493878 PMC6076953 30105267

[24] Shih JC, Liu KL, Shyu MK (2005). Temporary balloon occlusion of the common iliac artery: new approach to bleeding control during cesarean hysterectomy for placenta percreta. Am J Obstet Gynecol.

[25] David A, Hicham AZ, Malak M (2020). etal.“You only live twice”: multidisciplinary management of catastrophic case in placenta Accreta Spectrum-a case report. BMC Pregnancy Childbirth.

